# Oral treatment with *Eubacterium hallii* improves insulin sensitivity in *db/db* mice

**DOI:** 10.1038/npjbiofilms.2016.9

**Published:** 2016-07-06

**Authors:** Shanthadevi Udayappan, Louise Manneras-Holm, Alice Chaplin-Scott, Clara Belzer, Hilde Herrema, Geesje M Dallinga-Thie, Silvia H Duncan, Erik S G Stroes, Albert K Groen, Harry J Flint, Fredrik Backhed, Willem M de Vos, Max Nieuwdorp

**Affiliations:** 1 Department of Vascular Medicine, Academic Medical Center, Amsterdam, The Netherlands; 2 Wallenberg Laboratory, University of Gothenburg, Gothenburg, Sweden; 3 Laboratory of Microbiology, Wageningen University, Wageningen, The Netherlands; 4 Microbiology Group, Rowett Institute for Nutrition and Health, University of Aberdeen, Aberdeen, UK; 5 Department of Pediatrics, Laboratory of Metabolic Diseases, Groningen, The Netherlands; 6 Novo Nordisk Foundation Center for Basic Metabolic Research, Section for Metabolic Receptology and Enteroendocrinology, Faculty of Health Sciences, University of Copenhagen, Copenhagen, Denmark; 7 RPU Immunobiology, Department of Bacteriology and Immunology, Faculty of Medicine, University of Helsinki, Helsinki, Finland; 8 Diabetes Center, Department of Internal medicine, VU University Medical Center, Amsterdam, The Netherlands; 9 ICAR, VU University Medical Center, Amsterdam, The Netherlands

## Abstract

An altered intestinal microbiota composition is associated with insulin resistance and type 2 diabetes mellitus. We previously identified increased intestinal levels of *Eubacterium hallii*, an anaerobic bacterium belonging to the butyrate-producing *Lachnospiraceae* family, in metabolic syndrome subjects who received a faecal transplant from a lean donor. To further assess the effects of *E. hallii* on insulin sensitivity, we orally treated obese and diabetic *db/db* mice with alive *E. hallii* and glycerol or heat-inactive *E. hallii* as control. Insulin tolerance tests and hyperinsulinemic-euglycemic clamp experiments revealed that alive *E. hallii* treatment improved insulin sensitivity compared control treatment. In addition, *E. hallii* treatment increased energy expenditure in *db/db* mice. Active *E. hallii* treatment was found to increase faecal butyrate concentrations and to modify bile acid metabolism compared with heat-inactivated controls. Our data suggest that *E. hallii* administration potentially alters the function of the intestinal microbiome and that microbial metabolites may contribute to the improved metabolic phenotype.

## Introduction

The prevalence of obesity and type 2 diabetes mellitus is expected to rise to 1 in 3 adult subjects having type 2 diabetes mellitus in 2050.^1^ The pathophysiology of these metabolic disorders is complex, involving both environmental (dietary) and genetic factors affecting altered intestinal microbiota composition.^2^ Insulin resistant subjects are characterised by reduced levels of short-chain fatty acid (SCFA)-producing bacteria.^3,4^ Moreover, daily oral supplementation with the SCFA butyrate exerts beneficial effects on insulin resistance and dyslipidemia in diet-induced obese mice.^5^ Transplantation of lean healthy microbiota in both murine and human models of insulin resistance has been shown to significantly improve insulin sensitivity and to increase levels of butyrate-producing bacteria in the gut.^6,7^ With regards to the latter, we identified a specific increase in the butyrate-producer *Eubacterium hallii* in small intestinal biopsies of human obese and insulin resistant subjects upon lean donor faecal transplantation,^7^ which was associated with improved (peripheral) insulin sensitivity.

*E. hallii* is an anaerobic, Gram-positive, catalase-negative bacterium belonging to the *Lachnospiraceae* family of the phylum *Firmicutes* that is present in both murine and human faeces.^8^ *E. hallii* is a butyrate-producing species. Interestingly, in contrast to other intestinal bacterial isolates like *Roseburia* and *Faecalibacterium* that produce butyrate from monosaccharides, *E. hallii* has the capacity to also produce butyrate from lactate and acetate in a low pH environment such as the proximal small intestine.^9^ However, *in vivo* treatment with oral *E. hallii* has never been performed. We therefore performed a study in obese and insulin resistant *db/db* mice to investigate whether oral administration (by gavage) of *E. hallii* would have beneficial effects on insulin sensitivity. Upon identification of the optimal *E. hallii* treatment dose of 10^8^ CFU per day, we used this dose to subsequently investigate the effect of active and heat-inactivated *E. hallii* treatment on insulin sensitivity and energy metabolism using hyperinsulinemic-euglycemic clamp and metabolic cage approaches.

We found that oral treatment with active *E. hallii* improved insulin sensitivity in severely insulin resistant *db/db* mice and significantly increased energy expenditure. Furthermore, our data indicate that *E. hallii* mildly modifies SCFA production and bile acid composition, which potentially contributes to the beneficial effects of *E. hallii* treatment on insulin sensitivity in obese and diabetic *db/db* mice.

## Results

### *E. hallii* treatment dose-dependently improves insulin-mediated glucose clearance

**Figure 1:**
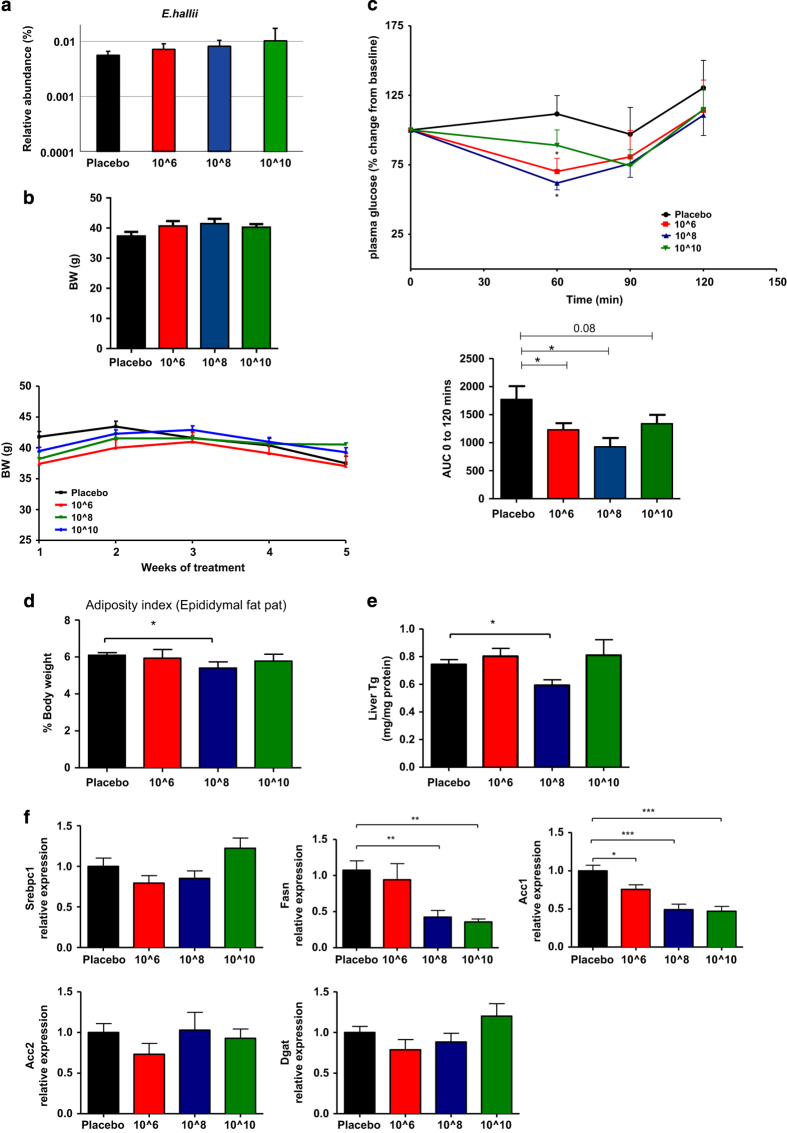
*E. hallii* treatment dose-dependently improves insulin sensitivity. Male *db/db* mice (*n*=8 per group) were daily treated with vehicle or increasing doses of *E. hallii* by gavage for 4 weeks. Figures depict effect of *E. hallii* treatment on (**a**) relative abundance of *E. hallii* in caecum, (**b**) body weight (showing average body weight per treatment group after 4 weeks of treatment and weekly weight gain throughout treatment period), (**c**) insulin tolerance test (showing insulin-mediated glucose clearance on *t*=60, 90 and 120 min after insulin administration and corresponding area under the curve (AUC)), (**d**) adiposity index (epididymal fat pad as % of body weight), (**e**) hepatic triglyceride (TG) content, (**f**) expression levels of hepatic lipogenic genes. Data are mean±s.d. Statistical analysis was performed using Student’s *t-*test. *P<0.05; **P<0.01.

Oral butyrate supplementation has been previously reported to regulate insulin sensitivity.^5^ As *E. hallii* is a butyrate-producing bacterium, we assessed whether administration of *E. hallii* could have beneficial effects on insulin sensitivity in a mouse model for diabetes. We therefore explored the effects of oral administration of increasing dosages of *E. hallii* on basal parameters (i.e., body weight and food intake) and insulin responsiveness in severely obese and diabetic *db/db* mice. We found a dose-dependent increase in caecal *E hallii* concentrations upon treatment with 100 μl of active 10^6^, 10^8^ and 10^10^CFU *E. hallii* (once daily for four weeks) (Figure 1a). Nevertheless, global analysis showed no major effect on the intestinal communities (data not shown). Importantly, body weight remained stable in all treatment groups compared with glycerol-treated controls (10^6^ CFU: 38±1.5 g, 10^8^ CFU: 40±0.3 g and 10^10^ CFU: 41±0.3 g versus placebo: 39±1.3 g. NS; Figure 1b). We then set out to assess insulin responsiveness by performing intraperitoneal insulin tolerance tests (ITT) in all treatment groups. Interestingly, *E. hallii*-treated groups displayed significantly improved insulin-mediated reduction in blood glucose levels (10^6^ CFU: −32±7%, 10^8^ CFU: −39±9% and 10^10^ CFU; −34±7% *P*<0.05) after 4 weeks of treatment compared with glycerol-treated controls (−2±7% *P*<0.05; Figure 1c). Altogether, these data indicate that *E. hallii* treatment improves insulin-mediated reduction in glucose levels without affecting food intake and body weight in severely obese and diabetic mice. The 10^8^ CFU *E. hallii*-treated mice exhibited the most remarkable response to insulin at all time points (*t*=60, 90 and 120 min). In addition, 10^8^ CFU *E. hallii* administration resulted in significantly reduced epididymal fat pad weight (Figure 1d) and hepatic triglyceride levels (Figure 1e), which was also reflected in the expression pattern of genes involved in lipogenesis (*Fasn* and *Acc1* were significantly reduced, (Figure 1f) and gluconeogenesis (trend towards reduction of *G6Pc, Pk, Pck1* were noticed), (Supplementary Figure S1). This to us suggested that 10^8^ CFU of *E. hallii* would be the optimal dosage to perform further investigations.

### *E. hallii* treatment improves insulin sensitivity and increases energy expenditure

**Figure 2:**
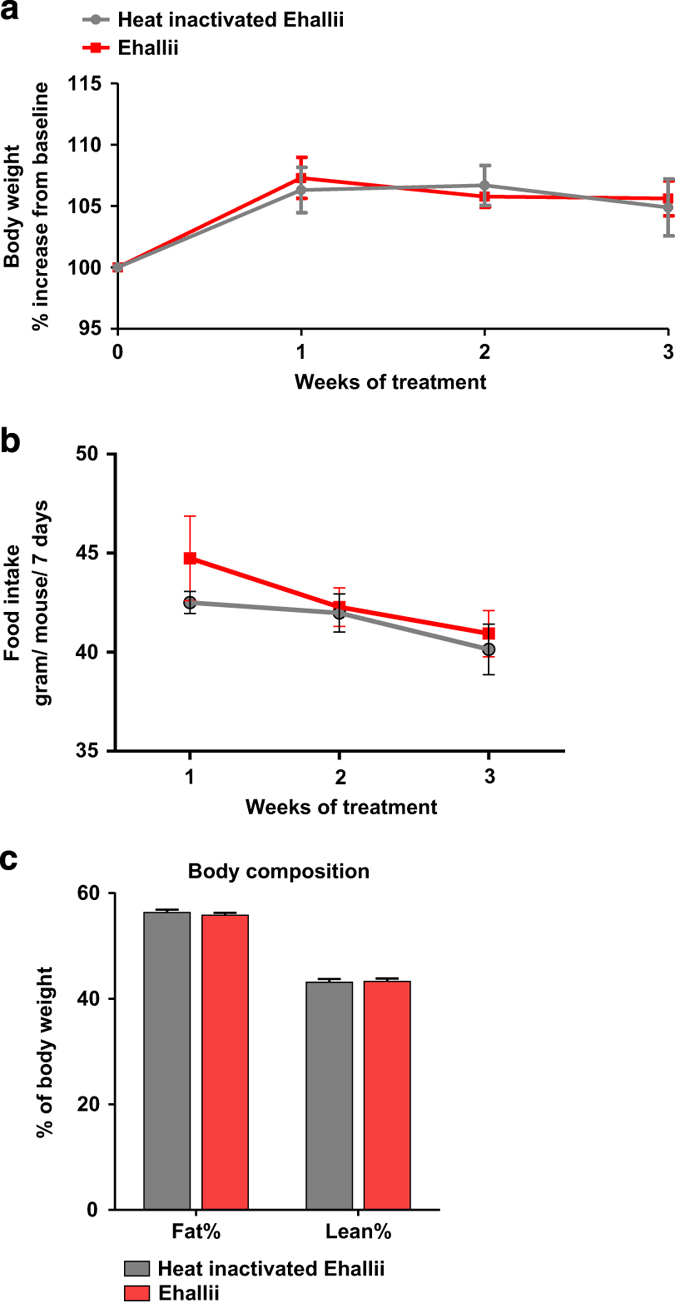
Effect of active and heat-inactivated *E. hallii* on body weight, food intake and body composition. Male *db/db* mice (*n*=7–10 per group) were daily treated with active or heat-inactivated *E. hallii* (10^8^ CFU) for 4 weeks. Figures depict effect of active or heat-inactivated *E. hallii* treatment on (**a**) body weight. (**b**) food intake and (**c**) body composition (as determined by magnetic resonance imaging). Data are mean±s.d. Statistical analysis was performed using Student’s *t-*test *P<0.05.

On the basis of the results from the dose–response study, we chose 10^8^ CFU *E. hallii* as daily therapeutic dose and repeated the study using active and heat-inactivated *E. hallii* as control. In line with observations from the dose–response study, body weight and food intake (Figure 2a,b) were similar in active and heat-inactivated *E. hallii*-treated mice. In addition, lean and fat mass (as % of body weight) were similar in both treatment groups (Figure 2c).

**Figure 3:**
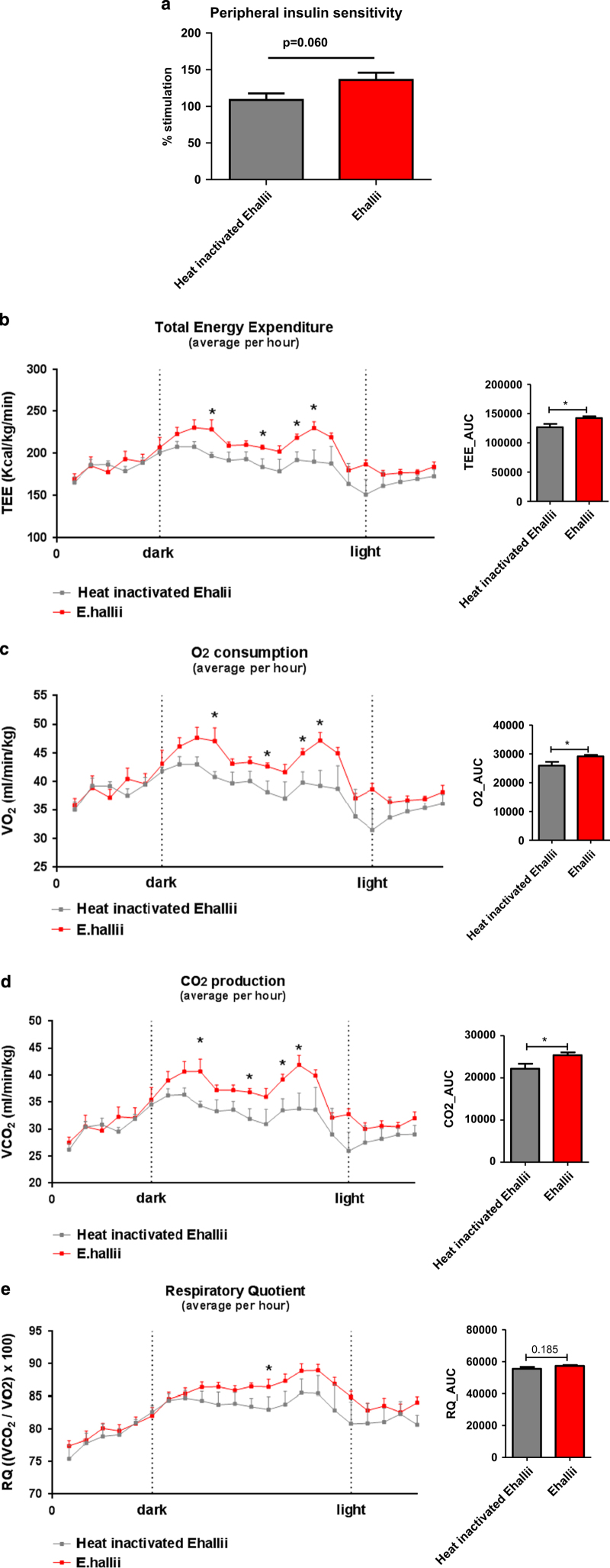
*E. hallii* treatment improves insulin sensitivity and energy expenditure. Male *db/db* mice (*n*=7–10 per group) were daily treated with active or heat-inactivated *E hallii* (10^8^ CFU) for 4 weeks. Figures depict (**a**) effect on peripheral insulin sensitivity as assessed by hyperinsulinemic-euglycemic clamp, (**b**) total energy expenditure, (**c**) O_2_ consumption, (**d**) CO_2_ production and (**e**) respiratory exchange ratio. Data are mean±s.d. Statistical analysis was performed using Student’s *t-*test *P<0.05.

Considering the effects of *E. hallii* treatment on insulin-mediated reduction in glucose levels as assessed by ITT, we moved forward with an in-depth assessment of insulin sensitivity by performing hyperinsulinemic-euglycemic clamp experiments in conscious, unrestrained mice. We assessed the ability of insulin to suppress endogenous Ra (endogenous rate of appearance, a marker of hepatic glucose production) and whole-body glucose disappearance (Rd; Supplementary Table S1). Although endogenous glucose production was not significantly altered (active: −33.9±3.7% versus heat-inactivated: −41.1±5.4%, *P*=0.299), treatment with *E. hallii* led to a close-to-significant increase in the ability of insulin to stimulate Rd (active: 136% versus heat-inactivated: 109%, *P*=0.060; Figure 3a). Considering the fact that *db/db* mice are severely insulin resistant, the improved Rd following 4 weeks of *E. hallii* treatment is of significant biological relevance.

Butyrate supplementation has previously been shown to improve energy expenditure in diet-induced obese mice.^5^ Altogether with our data on the beneficial effects of *E. hallii*, a butyrate producer, on insulin sensitivity in *db/db* mice, this motivated us to assess the effect of *E. hallii* on energy expenditure in this mouse model. Energy expenditure, oxygen consumption and CO_2_ production were monitored in metabolic chambers. Interestingly, active *E. hallii* treatment significantly increased total energy expenditure (active: 214±4 kcal/kg/min versus heat-inactivated: 191±9 kcal/kg/min, *P*<0.05; Figure 3b), oxygen consumption (active: 44.1±0.9 ml/min/kg versus heat-inactivated: 39.6±1.8 ml/min/kg, *P*<0.05; Figure 3c) and CO_2_ production (active: 38.0±1.0 ml/min/kg versus heat-inactivated: 33.4±1.8 ml/min/kg, *P*<0.05; Figure 3d) in the dark cycle. Respiratory quotient (expressed as *V*CO_2_/*V*O_2_) was not significantly altered (Figure 3e). In addition, to assess potential *E. hallii*-mediated changes in energy absorption, we analysed genes involved in glucose and lipid absorption in proximal part of the intestine. *E. hallii* treatment reduced intestinal genes involved in glucose (*Sglt1* and *Glut2*) transport and lipid absorption (*Cd36* and *Fatp4*; Supplementary Figure S2).

**Figure 4:**
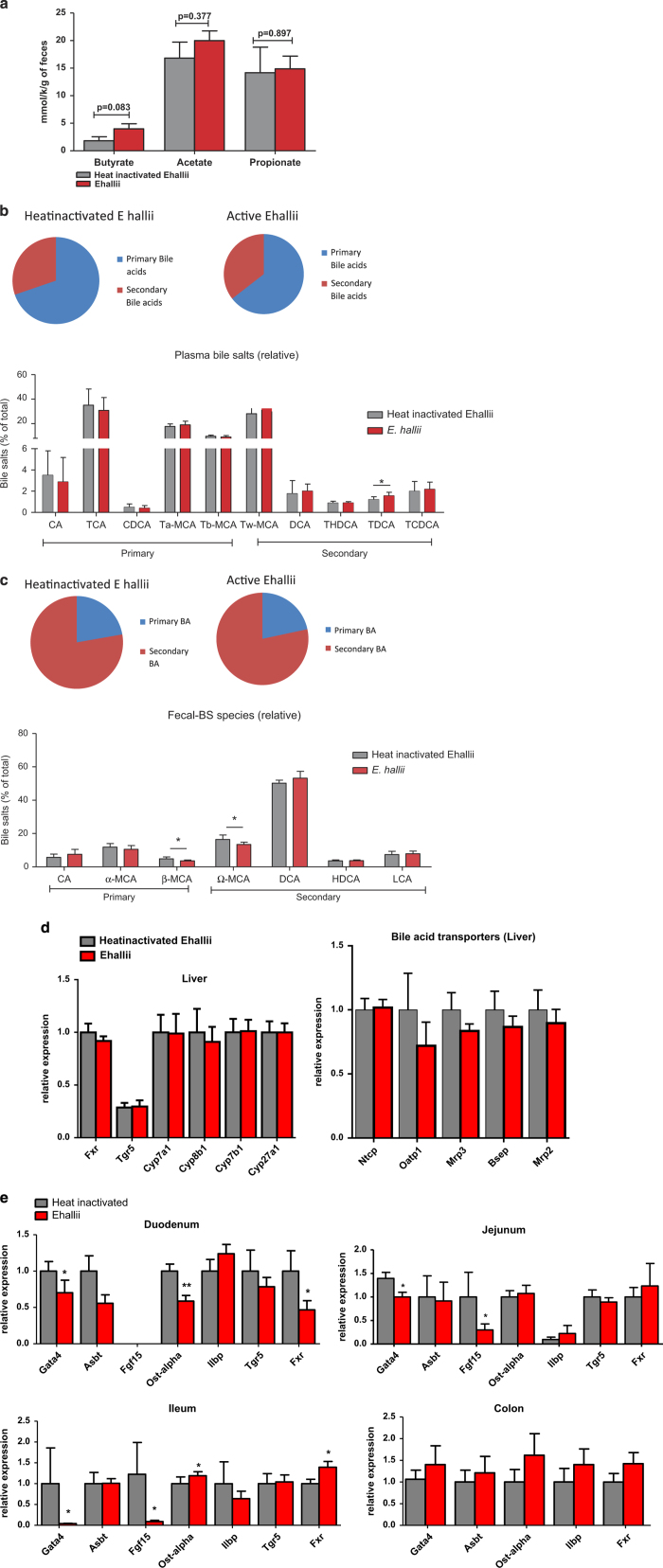
Effect of *E. hallii* treatment on short-chain fatty acids (SCFA’s) and bile acids. Male *db/db* mice (*n*=7–10 per group) were daily treated with active or heat-inactivated *E. hallii* (10^8^ CFU) for 4 weeks. Figures depict (**a**) faecal SCFA levels, (**b**) plasma primary and secondary bile acids and plasma bile acid composition, (**c**) faecal primary and secondary bile acids and plasma bile acid composition, (**d**) hepatic and (**e**) intestinal (duodenum, jejunum, ileum and colon) expression of genes involved in bile acid metabolism and transport. Data are mean±s.d. Statistical analysis was performed using Student’s *t-*test *P<0.05; **P<0.01.

To assess whether treatment with *E. hallii* increased SCFA levels, potentially providing insight into *E. hallii*-mediated effects on energy metabolism, we collected faeces (24 h) and measured concentrations of the SCFA’s butyrate, acetate and propionate. Active *E. hallii* treatment moderately increased faecal butyrate concentrations compared with heat-inactivated controls while propionate and acetate concentrations remained unaffected (Figure 4a).

Alterations in gut microbiota composition have significant impact on bile acid levels and bile acid composition.^10^ In addition to their role in solubilising food and uptake of food-soluble vitamins, bile acids are also important regulators of glucose and energy homeostasis.^11^ We therefore assessed whether active *E. hallii* treatment affected plasma and faecal bile acid levels and composition. Plasma primary and secondary bile acid levels were similar in active versus heat-inactivated *E. hallii-*treated mice (Figure 4b, pie chart). Further analysis of primary and secondary bile acid species revealed that the concentration of the secondary bile acid tauro-conjugated deoxycholic acid was significantly increased (Figure 4b, bar graph). Faecal primary and secondary bile acid levels remained unaffected by active *E. hallii* treatment (Figure 4c, pie chart). Levels of the primary bile acid β-MCA and the secondary bile acid ω-MCA, however, were significantly reduced in active versus heat-inactivated *E. hallii* -treated mice (Figure 4c, bar graph).

We then assessed expression levels of genes involved in bile acid metabolism and transport in liver and small intestine. Bile acid synthesis is tightly regulated by the bile acid receptor Farnesoid X receptor (*Fxr*) in liver and intestine. Hepatic *Fxr* exerts negative feedback control on cholesterol 7 alpha-hydroxylase (*Cyp7al*), the rate-limiting enzyme in hepatic bile salt synthesis.^12^ Expression levels of hepatic *Fxr* and *Cyp7a1* were similar in active and heat-inactive *E. hallii*-treated *db/db* mice (Figure 4d). Expression of genes encoding bile acid-synthetic genes such as (*Cyp7a1, Cyp8b1, Cyp7b1 and Cyp27a1*) and bile acid transporters (*Ntcp, Oatp1, Mrp3, Bsep and Mrp2*) remained unaffected by active *E. hallii* treatment. Although expression of *Fxr* in the small intestine was not altered by active *E. hallii* treatment, levels of fibroblast growth factor 15 (*Fgf15*), an FXR-target gene, were significantly reduced, which is suggestive of reduced activation of FXR in the intestine (31). We investigated the effect of active *E. hallii* treatment on genes regulating intestinal bile acid absorption by analysing the transcription factor (*Gata4*), apical sodium-dependent bile acid transporter (*Abst*), apical organic solute transporter (*Ostα*) and ileal lipid binding protein *(Ilbp*).^13^ Active E. *hallii* treatment significantly reduced and increased the expression of *Gata4* and *Ostα,* respectively, compared with heat-inactive *E. hallii* treatment. Nevertheless, expression levels of *Ilbp* and *Abst* remained unaffected (Figure 4e).

## Discussion

The current study demonstrates that daily oral administration of *E. hallii* improves insulin sensitivity and increases energy metabolism in severely obese and diabetic *db/db* mice. Our observations that administration of increasing dosages of *E. hallii* did not affect body weight or food intake indicate that *E. hallii* treatment might be a safe and effective new probiotic strain to improve insulin sensitivity.

In the dose–response study, we found that *E. hallii* treatment improved insulin sensitivity, yet the highest treatment dose had less effect on insulin sensitivity than the lower dosages. This phenomenon was also found in a human intervention trial using *B. infantis* and might be explained by the fact that these high concentrations (>10^10^ CFU of bacterial strains) induce a crowding effect resulting in less efficient dispersion of the bacteria over the (small) intestine.^14^ Moreover, oral supplementation of heat-inactivated *E. hallii* had no effect on murine metabolism, which is in line with the previous data studying the role of specific microbial strains on insulin sensitivity.^15^ Moreover, as we did not see any effect on body weight and the fact that we do not have the data on locomotor activity upon 4 weeks of *E. hallii* treatment, further studies will have to elucidate the long-term effects of *E. hallii* on all these parameters.

It has long been recognised that intestinal bacteria affect SCFA concentrations.^8,9^ Bacterial fermentation of indigestible fibres in the intestine, for example, by the butyrate-producer *E. hallii*, results in the production of SCFAs such as butyrate. Oral SCFAs administration to mice fed a high-fat diet reduced body weight and improved insulin sensitivity without changing food intake or levels of physical activity.^5^ SCFAs have been suggested to act on food intake through G-protein-coupled receptors such as GPR41 and GPR43, which subsequently increase release of the satiety hormones PYY and GLP-1. Furthermore, butyrate has been implicated in regulation of intestinal gluconeogenesis thereby improving glucose and energy homeostasis.^16^ Although oral *E. hallii* treatment had only minor effects on intestinal *E. hallii* abundance, levels of the SCFA butyrate, a metabolite of *E. hallii*, were doubled (~217%, NS) in active *E. hallii*-treated mice compared with heat-inactivated *E. hallii*-treated controls. Increased butyrate levels might potentially mediate the observed beneficial effects on peripheral insulin sensitivity and energy expenditure in active *E. hallii*-treated *db/db* mice. However, this hypothesis would require further analysis.

After release into the duodenum, bile acids travel the length of the small intestine and are reabsorbed and transported back to the liver mainly in the distal ileum.^17,18^ *Ruminococcaceae* and *Lachnospiraceae* families of the *Firmicutes* phylum (such as *E. hallii*) can mediate primary bile acid conversion to secondary bile acids.^19^ Furthermore, modulation of intrinsic bacterial bile acid hydrolysis significantly impacts bile acid composition and subsequent metabolic processes in the host.^20^ Although we found only a small effect of *E. hallii* treatment on intestinal bile acid metabolism, it is tempting to speculate that *E. hallii* indeed affects energy metabolism and insulin sensitivity via bile acids. Indeed, the *E. hallii* L2–7 genome contains 2 complete functional bile salt hydrolase (*BSH*) genes (W.M.d.V., personal communication) and their role in bile acid metabolism is currently under detailed investigation. Although total plasma and faecal secondary bile acid levels were similar in active and heat-inactivated *E. hallii*-treated *db/db* mice, active *E. hallii* treatment increased levels of the secondary bile acid tauro-conjugated deoxycholic acid. Interestingly expression of other FXR targets such as *Ilbp* and *Abst* remained unaltered, but expression of the transcription factor *Gata4* decreased significantly. A similar association between *Gata4* and *Fgf15* was recently reported by Out *et al.*^21^ and might be a direct interaction of microbiota with *Gata4* expression as also suggested in (ref. 13). Furthermore, changes in intestinal bacteria have been shown to primarily affect *Fxr* targets in the small intestine and not the liver.^19,22,23^ This is in line with our observation of decreased expression of the intestinal *Fxr* target gene *Fgf15* but not the hepatic *Fxr* target *Shp* after active *E. hallii* treatment. Microbiota modifications using probiotics have been reported to facilitate changes in intestinal bile acid transport,^24^ which is in line with the appreciable elevation of the bile acid transporter *Ostα* in the present study.

In conclusion, we show that daily treatment for 4 weeks with *E. hallii* L2–7 has no adverse effects and exerts beneficial effects on metabolism, potentially via alterations in butyrate formation and bile acid and metabolism.^25,26^ Our data thus underscore the therapeutic potential of replenishing missing intestinal bacterial strains for the treatment of human insulin resistance.^27^ Further research to confirm optimal dose and long-term effects of *E. hallii* on human insulin sensitivity and bile acid metabolism is urgently awaited.

## Materials and methods

### *E. hallii* culture

*E. hallii* strain L2–7 was cultured under anaerobic conditions as described previously.^8,9^ Purity was identified by cellular morphology and 16S RNA gene sequence analysis. Cultures were grown to the end of the exponential phase, concentrated by anaerobic centrifugation, washed with phosphate-buffered saline, diluted in a solution containing maltodextrin and glucose in 10% glycerol until final concentrations of 10^6^ colony forming units (CFU), 10^8^ CFU and 10^10^ CFU in 100 μl were reached. Viability was assessed by most probable number analysis by dilution to extinction and confirmed by microscopic analysis. Samples were stored at −80 °C and used within 6 months during which viability was not noticeably affected.

### Animals

All animal experiment were conducted in accordance with the principles of the ‘Guide to the Care and Use of Experimental Animals’ and were approved by the local Animal Ethics Committee, Academic Medical Center-University of Amsterdam, and the University of Gothenburg Animal Studies Committee.

The methods were carried out in accordance with the approved guidelines. Male C57Bl6/J *db/db* mice (12 weeks old) were purchased from the Jackson Laboratories USA. Animals were housed at AMC SPF vivarium in groups of 5 animals/cage and fed *ad libitum* with regular chow diet (Research Diets, Inc.) and water. Mice were housed under constant temperature and a 12-h light/dark cycle. At 16 weeks of age, the animals were daily given an oral 100 μl gavage of comprising 10^6^, 10^8^ and 10^10^ CFU of *E. hallii* in 10% glycerol stock for 4 weeks (*n*=8 mice per group). As a control, an oral 100 μl gavage of 10% glycerol in phosphate-buffered saline was used (*n*=8 mice). Twenty-four-hour faeces were collected after 4 weeks of treatment (24 h collection) for bile acid composition analysis. In the last week of treatment and after an overnight fast, mice (*n*=8 per group) received an intraperitoneal insulin bolus (Actrapid 0.75 U/kg body weight) and blood glucose was measured (Ascensia Elite glucose meter, Bayer, Leverkusen, Germany) at *t*=0, 60, 90 and 120-min post injection for determination of insulin sensitivity. Thereafter, animals were sacrificed using 100 mg/kg pentobarbital and faeces and caecum were collected.

### Hyperinsulinemic-euglycemic clamp

Male C57Bl6/J6 *db/db* mice (12 weeks old) were purchased from the Jackson Laboratories, Bar Harbor, ME, USA. Animals were housed at University of Gothenburg SPF vivarium and fed *ad libitum* with regular chow diet (Research Diets, New Brunswick, NJ, USA) and water. Mice were housed under constant temperature and a 12-h light/dark cycle and underwent daily oral 100 μl gavage for 4 weeks with 10^8^ CFU active or heat-inactivated *E. hallii* (15 min at 70 °C) as control (*n*=7–10 per group). In the last week of treatment, at least 4 days before the clamp a catheter was surgically placed in the jugular vein for infusion of insulin and glucose under isoflurane anaesthesia. Prior to the clamp, mice were fasted for 4 h and placed in individual plastic containers. Basal blood glucose (Countour Next blood glucose meter, Bayer AB, Solna, Sweden) was used from tail-blood measurements. A bolus injection of [3-^3^H] glucose (5 μCi; PerkinElmer, Waltham, MA, USA) was given through the jugular vein catheter (*t*=−80 min prior insulin infusion), followed by a continuous infusion of 0.05 μCi/min for assessment of basal glucose turnover rate. Three consecutive blood samples were taken at steady state (*t*=−20, −10 and 0 min prior insulin infusion) for the determination of both plasma [3-^3^H] glucose and glucose concentration. At *t*=0, a priming dose of insulin (178 mU/kg; Actrapid Penfill, Novo Nordisk, Bagsværd, Denmark) was given, followed by a continuous insulin infusion rate of 20 mU/min/kg. The infusion of [3-^3^H] glucose was increased to 0.1 μCi/min during clamp to minimise changes in specific activity during insulin infusion. Blood glucose was measured at 10-min intervals, via tail-blood sampling, to adjust the glucose infusion rate (GIR; 30% glucose Fresenius Kabi, Bad Homburg, Germany) to maintain blood glucose concentration at the basal level. At steady state, defined by stable glycemia and GIR (approximately at *t*=120 m interval), three consecutive blood samples were taken at 10 min intervals to determine whole-body glucose utilisation (Rd) and hepatic glucose production (Ra) under hyperinsulinemic-euglycemic- conditions. Plasma insulin was measured at *t*=−10 min (basal) and 120 min (clamp). Animals were killed by an overdose of pentobarbital (Apoteket Farmaci AB, Stockholm, Sweden) and tissue was collected. The blood samples were deproteinised, evaporated and resuspended in deionised water for the determination of radioactivity (Beckman LS6500 Multipurpose Scintillation Counter, Providence, RI, USA). Whole-body glucose appearance (Ra) and endogenous glucose production (endogenous Ra), a measure of hepatic glucose production, were calculated as published as previously described.^28^

### Metabolic chamber experiments and body composition

During a parallel experiment, male *db/db* mice (aged 12 weeks, *n*=7–10 per group) were treated orally with 100 μl of 10^8^ CFU active or heat-inactivated *E. hallii* (15 min at 70 °C) as control for 4 weeks. Thereafter, mice were individually housed in Somedic INCA metabolic cages (Somedic AB, Hörby, Sweden) to study total energy expenditure and respiratory quotient. Oxygen consumption (*V*O_2_) and CO_2_ production (*V*CO_2_) were recorded every 2 min for 23 h. Temperature in the metabolic chamber was kept constant at 21 °C and animals had free access to food and water. Data from the first hour was discarded to account for animal acclimatisation. The average total energy expenditure per hour was determined using Weir's equation: (3.9×*V*O_2_)+(1.1×*V*CO_2_) and respiratory quotient was calculated as the *V*CO_2_/*V*O_2_ ratio. Also, magnetic resonance imaging scanning for body composition was performed as previously described.^25^

### Intestinal microbiota analysis

Abundances of *E. hallii* were determined in caecal content by using the Mouse Intestine Tract Chip as previously reported.^15,29^ Total genomic DNA was extracted from the frozen caecum with the QIAamp DNA stool mini-kit (Qiagen, Valencia, CA, USA) according to the manufacturer’s protocol. 16S rRNA gene amplification, *in vitro* transcription and labelling, and hybridisation were carried out as described.^30^ The data were normalised and analysed using a set of R-based scripts in combination with a custom-designed relational database, which operates under the MySQL database management system. For the microbial profiling, the Robust Probabilistic Averaging signal intensities of 2667 specific probes for the 94 genus-level bacterial groups detected on the MITChip were used.^31^ Diversity calculations were performed using a microbiome R-script package (https://github.com/microbiome). Multivariate statistics, redundancy analysis, and principal response curves were performed in Canoco 5.0 and visualised in triplots or a principal response curves plots.^32^

### SCFA and bile acid profiling

Twenty-four-hour faecal samples (pooled from each cage) were collected and stored for later analysis. SFCA content was analysed by gas liquid chromatography following conversion to *t*-butylmethylsilyl derivate as previously described.^9^ Concentrations of different bile acids were measured twice in 24-h faecal samples collected in week 4 and in plasma. An internal standard was added before extraction with 0.2 mol/l NaOH at 800 °C for 20 min. Bile salt were trimethylsilylated with pyridine, *N,O*-Bis(trimethylsilyl) trifluoroacetamide and trimethylchlorosilane. Faecal bile acid profile was measured using capillary gas chromatography (Hewlett–Packert gas chromatograph; HP 6890, Mountain View, CA, USA) equipped with a FID and a CP Sil 19 capillary column; length 25 m, internal diameter 250 μm and a film thickness of 0.2 μm (Chrompack BV, Middelburg, The Netherlands). Plasma bile acids were determined using liquid chromatography tandem mass spectrometry as described previously (17). The primary bile acids cholic acid (CA), taurocholic acid, muricholic acid (MCA), tauroalpha muricholic acid and taurobeta muricholic acid as well as the secondary bile acids taurohyodeoxycholic acid, deoxycholic acid, taurodeoxycholic acid and omega murocholic acid were analysed in plasma and 24 faeces.^21^ The total amount of primary and secondary bile acids was calculated as the sum of the individually quantified bile salts.

### Quantitative real-time PCR

Liver and intestinal tissues were homogenised with tissue-magnaLyzer (Roche, Basel, Switzerland). Total RNA was extracted using Tri-pure reagent (Roche). Complementary DNA was prepared by reverse transcription of 1 μg total RNA using reverse transcription kit (BioRad, Hercules, CA, USA). Hepatic genes involved in lipogenesis (*Srebp1c, Fasn, Acc1, Acc2* and *Dgat*) and gluconeogenesis (*Gck1, G6Pc, Pk* and *Pck1*) were examined. Genes involved in bile acid metabolism and transport were tested in liver (*Cyp7a1, Cyp8b1, Cyp7b1* and *Cyp27a, Ntcp, Oatp1, Mrp3, Bsep* and *Mrp2*) and proximal to distal intestinal segments (duodenum, jejunum and ileum) for (*Tgr5, Fxr, Gata4, Asbt, Ilbp Ostα* and *Fgf15*).^33^ Real-time quantitative PCR was performed using Sensifast SYBR master mix (GC biotech, Alphen a/d Rijn, the Netherlands). Gene-specific intron–exon boundary spanning primers were used and all the results were normalised with the house keeping gene *36B4*. All samples were analysed in duplicate and data were analysed according to the 2^ΔΔCT^ method. Primer sequences are presented in Supplementary Table S2.

### Statistical analysis

On the basis of distribution of the clinical data, Student *t*-test or Mann–Whitney tests (two-sided) were used to analyse the difference between clinical groups. Microbiota analyses were done as described above. *P value<0.05 or **P<0.01 were considered statistically significant.
