# Supplementary material for: Oral treatment with Eubacterium hallii improves insulin sensitivity in db/db mice
Source: NPJ Biofilms Microbiomes. 2016 Jul 6;2:16009–. doi: 10.1038/npjbiofilms.2016.9 (PMC5515273; doi:10.1038/npjbiofilms.2016.9)
Supplement: Supplementary Information [file npjbiofilms20169-s1.doc]

**Oral treatment with *Eubacterium hallii* improves insulin sensitivity in *db/db* mice**

Shanthadevi Udayappan (1), Louise Manneras-Holm (2), Alice Chaplin-Scott (1), Clara Belzer (3), Hilde Herrema (1), Geesje M. Dallinga-Thie (1), Silvia Duncan (4), Erik S. G. Stroes (1), Albert K. Groen (5), Harry Flint (4), Fredrik Backhed (2,6), Willem M. de Vos (3,7), Max Nieuwdorp (1,2,8,9)

(1) Department of Vascular Medicine, Academic Medical Center, Amsterdam, The Netherlands

(2) Wallenberg Laboratory, University of Gothenburg, Gothenburg, Sweden

(3) Laboratory of Microbiology, Wageningen University, Wageningen, The Netherlands

(4) Microbiology Group, Rowett Institute for Nutrition and Health, University of Aberdeen, Aberdeen, UK

(5) Department of Pediatrics, Laboratory of Metabolic Diseases, UMCG, Groningen, the Netherlands

(6) Novo Nordisk Foundation Center for Basic Metabolic Research, Section for Metabolic Receptology and Enteroendocrinology, Faculty of Health Sciences, University of Copenhagen, Copenhagen, DK-2200, Denmark.

(7) RPU Immunobiology, Department of Bacteriology and Immunology, Faculty of Medicine,, University of Helsinki, Helsinki, Finland

(8) Diabetes Center, Department of Internal medicine, VU University Medical Center, Amsterdam, The Netherlands

(9) ICAR, VU University Medical Center, Amsterdam, The Netherlands

*Corresponding author

M. Nieuwdorp, MD PhD

Department of Vascular Medicine

Academic Medical Center

Meibergdreef 9, room F4-159.2

1105 AZ Amsterdam, the Netherlands, email:m.nieuwdorp@amc.uva.nl

**Supplementary data**

**Supplementary Table 1. Parameters hyperinsulinemic euglycemic clamp**

|  | **HEAT-INACTIVATED** | **ACTIVE** | **P-value** |
| --- | --- | --- | --- |
| **Body weight (g)** | 48.1 ± 0.6 | 47.0 ± 0.7 | 0.266 |
| **Basal glucose (mM)** | 17.8 ± 2.5 | 19.6 ± 2.0 | 0.579 |
| **Clamp glucose (mM)** | 17.6 ± 2.6 | 19.9 ± 2.1 | 0.523 |
| **Basal insulin (ng/ml)** | 7.3 ± 0.8 | 6.0 ± 0.6 | 0.228 |
| **Clamp insulin (ng/ml)** | 138.3 ± 21.9 | 162.1 ± 28.4 | 0.524 |
| **GIR (mg/min/kg)** | 34.7 ± 2.3 | 36.6 ± 1.8 | 0.533 |
| **Rd basal (mg/min/kg)** | 23.3 ± 1.1 | 21.9 ± 1.1 | 0.381 |
| **Rd clamp (mg/min/kg)** | 48.6 ± 3.2 | 51.2 ± 2.2 | 0.524 |
| **EndoR_a_ (mg/min/kg)** | 13.9 ± 1.5 | 14.6 ± 1.2 | 0.748 |
| **% suppression Endo Ra** | -41.1 ± 5.4 | -33.9 ± 3.7 | 0.299 |

**Supplementary Table 2. Primer sequences for qPCR**

| **Gene** | **Abbreviation** | **Sequence** |
| --- | --- | --- |
| Apical sodium dependent bile acid transporter | Asbt | | TTGCCTCTTCGTCTACACC | | --- | | CCAAAGGAAACAGGAATAACAAG | |
| Acetyl-CoA carboxylase 1 | *Acc1* | GGGAGAAACAGGGAGGAAG  TCGAAAGTCACCCCGAATAG |
| Acetyl-CoA carboxylase 2 | *Acc2* | GGG CTC CCT GGA TGA CAA C  TTC CGG GAG GAG TTC TGG A |
| Bile salt export pump  (Abcb11) | *Bsep* | CTGCCAAGGATGCTAATGCA CGATGGCTACCCTTTGCTTCT |
| Cytochrome P450, family 7, subfamily A, polypeptide 1 (Cholesterol 7α- hydroxylase) | *Cyp7a1* | AGCAACTAAACAACCTGCCAGTACTA GTCCGGATATTCAAGGATGCA |
| Cytochrome P450, family 7, subfamily B, polypeptide 1 (Oxysterol 7α-hydroxylase) | *Cyp7b1* | TAGCCCTCTTTCCTCCACTCATA GAACCGATCGAACCTAAATTCCT |
| Cytochrome P450, family 8, subfamily B, polypeptide 1 (Sterol 12α-hydroxylase) | *Cyp8b1* | GGCTGGCTTCCTGAGCTTATT ACTTCCTGAACAGCTCATCGG |
| Cytochrome P450, family  27, subfamily A, polypeptide 1 (Sterol 27- hydroxylase) | *Cyp27a1* | GCCTCACCTATGGGATCTTCA TCAAAGCCTGACGCAGATG |
| Diglyceride acyltransferase2 | *Dgat2* | TTC CTG GCA TAA GGC CCT ATT  AGT CTA TGG TGT CTC GGT TGA C |
| Fibroblast growth factor 15 | *Fgf15* | ACGTCCTTGATGGCAATCG GAGGACCAAAACGAACGAAAT T |
| Farnesoid X receptor | *Fxr* | TCCAGGGTTTCAGACACTGG GCCGAACGAAGAAACATGG |
| Fatty acid synthase | *Fasn* | GCTGCTGTTGGAAGTCAGC  AGTGTTCGTTCCTCGGAGTG |
| Globin Transcription Factor4 | Gata4 | | AAGACACCCCAATCTCGATATGTT | | --- | | CATGGCCCCACAATTGACAC | |
| Glucokinase 1 | *Gck1* | TATGAAGACCGCCAATGTGA  TTTCCGCCAATGATCTTTTC |
| Glucose 6 phosphatase | *G6pc* | CCG GTG TTT GAA CGT CAT CT  CAA TGC CTG ACA AGA CTC CA |
| Ileal lipid-binding protein | Ilbp | | TGGCAAAGAATGTGAAATG | | --- | | CTCCGAAGTCTGGTGATAG | |
| Multidrug resistance- associated protein (Abcc2) | *Mrp2* | GGATGGTGACTGTGGGCTGAT GGCTGTTCTCCCTTCTCATGG |
| Multidrug resistance- associated protein (Abcc3) | *Mrp3* | TCCCACTTTTCGGAGACAGTAAC ACTGAGGACCTTGAAGTCTTGGA |
| Na^+^/taurocholate  cotransporter | *Ntcp* | ATGACCACCTGCTCCAGCTT GCCTTTGTAGGGCACCTTGT |
| Organic anion transporting protein 1 | *Oatp1* | CAGTCTTACGAGTGTGCTCCAGAT ATGAGGAATACTGCCTCTGAAGTG |
| Organic solute transporter α | *Osta* | TGTTCCAGGTGCTTGTCATCC CCACTGTTAGCCAAGATGGAGAA |
| Phosphoenolpyruvate Carboxykinase 1 | *Pck1* | ATGTGTGGGCGATGACATT  AACCCGTTTTCTGGGTTGAT |
| Pyruvate kinase | *Pk* | TTCTGTCTCGCTACCGACCT  CCTGTCACCACAATCACCAG |
| Sterol regulatory element binding protein 1 | *Srebp1c* | GCAGACTCACTGCTGCTGAC  AGGTACTGTGGCCAAGATGG |
| G protein-coupled bile acid receptor 5 | *Tgr5* | | GCTCCTGTCAGTCTTGGCCTAT | | --- | | TTCCTCGAAGCACTCGTAGACA | |

**Supplementary Figure 1.** Male *db/db* mice (n = 8 per group) were daily treated with vehicle or increasing doses of *E hallii* by gavage for 4 weeks. Figure depicts effect of *E. hallii*-treatment on expression levels of hepatic glucose metabolic genes. Data are mean ± sd. Statistical analysis was performed using Student’s T test * p<0.05.

**Supplementary Figure 2.** Male *db/db* mice (n = 7-10 per group) were daily treated with active or heat-inactivated *E hallii* (10^8^ CFU) for 4 weeks. Figure depicts effect of *E. hallii*-treatment on expression levels of intestinal genes involved in glucose and lipid metabolism. Data are mean ± sd. Statistical analysis was performed using Student’s T test * p<0.05.

**Supplementary Figure 1:**

**Supplementary Figure 2:**
